# *Hermetia illucens* in the Process of Kitchen Waste Biodegradation: The Effect of Different Approaches to Waste Storage on the Microbiological Profile and Nutritional Parameters of the Larvae

**DOI:** 10.3390/insects16010087

**Published:** 2025-01-16

**Authors:** Zuzana Mašková, Juraj Medo, Eduard Kolesár, Dana Tančinová, Eva Ivanišová, Dana Urminská, Lukáš Hleba, Jana Urminská, Monika Mrvová, Zuzana Barboráková

**Affiliations:** 1 Institute of Biotechnology, Faculty of Biotechnology and Food Sciences, Slovak University of Agriculture in Nitra, Tr. A. Hlinku 2, 949 76 Nitra, Slovakia; juraj.medo@uniag.sk (J.M.); dana.tancinova@uniag.sk (D.T.); dana.urminska@uniag.sk (D.U.); lukas.hleba@uniag.sk (L.H.); xmrvova@uniag.sk (M.M.); zuzana.barborakova@uniag.sk (Z.B.); 2 ecol Trade, s.r.o., J. Haška 1, 949 01 Nitra, Slovakia; ecoltrade@gmail.com; 3 Institute of Food Sciences, Faculty of Biotechnology and Food Sciences, Slovak University of Agriculture in Nitra, Tr. A. Hlinku 2, 949 76 Nitra, Slovakia; eva.ivanisova@uniag.sk (E.I.); jana.urminska@uniag.sk (J.U.)

**Keywords:** bioconversion, black soldier fly, BSFL, food waste storage, *Hermetia illucens*, microbiological safety, protein and fat content

## Abstract

This study assessed the bioconversion efficiency of *Hermetia illucens* larvae (BSFL) fed on food waste stored under different conditions, focusing on the nutritional and microbial quality of the resulting larval biomass. Food waste was prepared as a fresh diet (FD) or naturally contaminated and stored at 20–22 °C (OS-T, opened storage-tempered) or under refrigeration, at 5–8 °C (CS-C, closed storage-cooled). Refrigerated, closed storage (CS-C) led to the highest rates of waste reduction (91.0%) and bioconversion efficiency (30.2%), with larvae exhibiting the highest protein content (36.83%) compared to the FD (35.5%) and OS-T (34.71%) groups. Microbiome analysis revealed that the CS-C condition promoted beneficial yeasts like *Pichia* and *Diutia*, which correlated positively with improved protein content and microbial safety. In contrast, OS-T storage supported spoilage fungi (*Mucor*, *Rhizopus*) and elevated total aerobic counts (7.28 log CFU/g), indicating higher microbial risks. The observed trends in waste reduction and protein content most probably relate to differences in microbial profiles, as controlled cooling affected microbial dynamics, preserving substrate quality and supporting larval growth. These findings emphasize the importance of refrigerated, closed storage to optimize bioconversion, improve larval nutritional value, and minimize microbiological hazards.

## Simple Summary

Food waste is increasing globally as living standards rise and the population grows. Effective management approaches are required to reduce environmental contamination. One growing alternative option is the bioconversion process, which treats organic waste using living organisms such as insects. Black soldier fly larvae (BSFL) can rapidly and effectively convert huge amounts of food waste into useful biomass, high in proteins and lipids, which can be utilized as animal feed or an alternative food source. Because microbiological safety and larval nutritional value are crucial, this study focused on how waste storage conditions affect these characteristics. The results showed that the waste handling method and its resulting quality significantly affect the microbial composition (load and diversity) and nutritional profile (protein, fat, and dry matter content) of BSFL. Waste storage in closed containers at refrigerator temperatures proved to be more suitable than open storage at room temperature, not only because of the improved microbial safety of the larvae but also because of a higher increase in the weight of the final larval biomass and accumulated protein content. This study suggests that proper waste handling methods can optimize the biodegradation process and improve BSFL quality and safety.

## 1. Introduction

Food waste (FW) is a significant component of global organic waste, with its volume rising due to population growth and higher living standards [1,2,3]. The Food and Agriculture Organization (FAO) estimates that 1.3 billion metric tons, or one-third of the world’s food supply, is wasted annually [4]. This waste contributes to greenhouse gas emissions from decomposition in landfills [5,6,7], with households in high-income countries generating over half of it [8]. Sustainable approaches, aligned with the circular economy, aim to reuse resources and transform waste into valuable products [9,10].

Bioconversion using insects, particularly black soldier fly larvae (*Hermetia illucens*, BSFL), is a promising solution for FW management [10,11]. BSFLs efficiently convert diverse organic substrates into protein- and lipid-rich biomass [12,13,14,15], while the residual frass can serve as a fertilizer [6,16]. This sustainable process is gaining attention for its potential in waste valorization [17,18,19,20], with applications in animal feed, aquaculture, and potential novel human foods [18,21,22,23].

Numerous studies have explored the effectiveness of BSFL in reducing FW and other organic waste. *H. illucens* larvae exhibit exceptional waste conversion efficiency, transforming up to 50% of the dry matter of FW into larval biomass [19,24]. Under optimal conditions, larvae can contain up to 40% protein and 30–35% fat in dry matter [12,13,15,19]. They are particularly effective in degrading nitrogen-rich and lipid-rich waste, including meat and dairy products, which are challenging to process through traditional methods like composting [15,20]. BSFLs are characterized by their high tolerance to various substrate conditions and their relatively rapid growth, which makes them a versatile tool in organic waste management [14,19]. Temperature and storage conditions of FW can significantly influence the quality of the resulting larval biomass. High temperatures or prolonged storage of FW can lead to microbial decomposition of organic matter, reducing the nutritional value of the substrate for larvae [25,26]. Conversely, appropriately stored FW, such as refrigerated or airtight conditions, can preserve nutrients and minimize microbial spoilage, leading to improved larval growth and protein accumulation [25,27].

The nutritional composition and microbiological safety of final BSFL biomass are key determinants of their usability. Current research highlights the significant impact of rearing substrates on these variables [25,28,29,30]. The microbiome of BSFL, including their gut- and cuticle-associated microorganisms, plays a pivotal role in enhancing digestion, breaking down organic matter, and influencing the overall bioconversion process [31,32]. Larval microbiomes are influenced by several factors, including the composition and storage conditions of the substrate [31,33]. Poorly managed FW, such as improperly stored kitchen waste, can harbor harmful microorganisms, including pathogens and mycotoxigenic fungi [29,34]. If the larvae are kept on contaminated substrate for an extended period, they may become contaminated themselves [35,36]. These microbes can either persist in the larvae or proliferate during the bioconversion process, posing potential risks to feed and food safety [37,38]. Studies on the microbiome of *H. illucescens* have mostly focused on gut bacteria and reveal that BSFL’s gut microbiota is primarily composed of bacterial genera like *Morganella*, *Providencia*, and *Enterococcus*, which are crucial in breaking down organic matter and generating nutrients [32,39]. But in terms of the microbiological safety of the larvae produced, the composition of the total microbiome is much more important. Microorganisms that proliferate during FW storage prior to bioconversion are even more pronounced in the final larval microbiome, including potentially dangerous genera [37]. The external microbiota usually reflects the microbial composition of the substrate and can include opportunistic pathogens like *Yersinia*, *Pantoea*, and *Acinetobacter*, particularly when the substrate is poorly managed [37,39]. Additionally, improperly stored FW can result in an overabundance of filamentous fungi, which could lower larval productivity and present further hazards because of the possible production of mycotoxins [34,40]. The microbial load of potential pathogens usually decreases during bioconversion, but the decrease could be inadequate for sufficient elimination [27]. As a result, one of the most serious biological risks linked with edible insects is the presence of pathogenic and toxigenic microorganisms [30]. Therefore, it is highly important to study the microbiomes of BSFL raised on a specific diet under specific conditions [41].

While BSFL bioconversion holds great promise, several challenges remain. The processes governing the microbiological changes in FW and their impact on larval microbiota are still not fully understood [36,39]. Further research is needed to optimize substrate storage conditions and develop cost-effective solutions for managing microbial risks associated with FW [27,42]. Addressing these challenges will help maximize the benefits of BSFL bioconversion while ensuring the microbiological safety of the resulting biomass.

The aim of the research was to analyze the effect of different food waste storage methods on the process of its bioconversion by *H. illucens* larvae. We focused on analyzing the nutritional variables of the larvae, specifically protein, fat, dry matter, and ash content, which are key indicators of their nutritional quality for use as animal feed and potential human food. Additionally, we evaluated the efficiency of the bioconversion process. In terms of microbiological safety, the aim was to find out what the microbiological load of the larvae is after the bioconversion process and what the composition of their total microbiome is. The findings can thus contribute to the optimization of the food waste management practices by providing insights into how different storage methods impact the nutritional quality and microbiological safety of BSFL.

## 2. Materials and Methods

### 2.1. Rearing of Larvae and Configuration of the Experiment

BSFL were obtained from experimental colonies established and reared under standard commercial conditions at ecol Trade s.r.o. (Nitra, Slovakia), a company dealing with the collection, transport, and disposal of biological waste. At the start of the experiment, the larvae were approximately 10 days old and had been reared on a standard chicken feed (a mixture of cereals, proteins, and fats, which provides a sufficient source of energy and nutrients).

Obtained larvae were bred in rooms without access to direct light, at a temperature of approximately 27 ± 1 °C (mean ± sd) and a relative humidity in the range 50–70%. The larvae were kept in plastic closable containers (radius 11 cm, height 23 cm, and volume 8.7 L) with a built-in metal insect net in the lid, which was used for ventilation. The air flow of the room was provided by standard stand fans. After 8 days of the experiment, BSFL were separated from the frass (residue) by sieving, followed by picking up the larvae with forceps, and were immediately analyzed (weight, count), killed by freezing at −80 °C, and analyses were then performed (protein, fat, microbial load, and microbiome).

### 2.2. Feed Formulation and Feeding Process

The feed was formulated to simulate kitchen waste (hydrated couscous, boiled egg, fresh spinach, and carrot peels in a ratio of 30:5:1:2) and designed in 3 variants. The first was a freshly prepared diet (FD), and another two variants were formulated to map the microbial load of perishable waste. Immediately after the preparation of the fresh feed, both variants were exposed to natural contamination by microorganisms from the environment in open containers for one full day. Subsequently, one container was closed and cooled to 5–8 °C for the next 5 days (closed storage-cooled, CS-C). Another variant remained for the following days at room temperature, 20–22 °C (open storage-tempered, OS-T). After incubation, the feeds of both variants showed signs of microbial action, as there were visible sensory changes. The OS-T diet showed discolorations and significant growth of microscopic filamentous fungi and a musty odor was noted. The CS-C diet showed altered odor and color, and was contaminated with filamentous fungi, but to a substantially lesser extent.

All experimental groups (variants) were performed in quadruplicate in separate containers maintained in parallel. Each container held 100 g of BSFL, approximately 3200 individuals, and received 1140 g of feed at the start of the experiment.

The moisture content (MC) and water activity (a_w_) of individual diet variants were assessed. The MC was determined by calculating the difference in weight of 5 g of the initial sample before and after drying in an oven for 4 h at 105 °C to a constant weight. A LabMaster-aw device (Novasina, Lachen, Switzerland) was used to measure a_w_.

### 2.3. Biometric and Nutritional Characteristics of Larvae

#### 2.3.1. Weigh

The average weight of the larvae before and after the experiment was evaluated as the mean weight of 100 individuals of every larval group (BSFL before the experiment and BSFL of all variants after the feeding process in all four replicates). The final weight of total larval biomass and the total weight of the residue were determined.

#### 2.3.2. Dry Matter and Ash Content

The dry matter of the input larvae, output larvae, diet samples, and residues were determined gravimetrically according to Ruiz [43]. Samples were dried to constant mass in an oven at 105 ± 5 °C, and the difference in mass before and after the drying process was used to determine the dry matter. The ash content was evaluated by incineration of the dried samples at 550 °C in a muffle furnace Nabertherm P 330 (Nabertherm GmbH, Lilienthal, Germany) until complete combustion.

#### 2.3.3. Protein Content

BSFL, 100 g of each sample, were lyophilized (Laboratory freeze-dryer Christ LyoCube Alpha 1-4 LSC plus, Osterode am Harz, Germany). Subsequently, 10 g of the larvae were homogenized with a blender. To 1 g of homogenate, 10 mL of demineralized water was added. Protein extraction was performed under constant stirring (Rocker shaker BioSan MR-12, Riga, Latvia) for 60 min at 20–22 °C. The extract was filtered through filter paper no. 390 (Munktell and Filtrak Gmbh, Bärenstein, Germany) and used to determine protein concentration according to the Bradford method [44].

The protein concentration was calculated using a calibration curve. The standard protein solution was prepared using bovine serum albumin (SERVA Electrophoresis GmbH, Heidelberg, Germany) at a concentration of 1 mg/mL in deionized water. The calibration curve expresses the dependence of the absorbance at 595 nm on the protein concentration in mg/mL.

#### 2.3.4. Fat Content

An Ancom XT15 Fat Extractor (Ancom, Macedon, NY, USA) device was used to detect the amount of fat in larval samples. According to the manufacturer’s instructions, a total of 1.5 g of sample was weighed (W1) into a special filter bag XT4 (Ancom, Macedon, NY, USA) and dried for 3 h at 105 °C to remove moisture before extraction. The samples were subsequently placed for 15 min in a bag with a desiccant, weighed again (W2), and then extracted with petroleum ether for 60 min at 90 °C. After this procedure, the samples were removed, dried in an oven for 30 min at a temperature of 105 °C, placed in a bag with a desiccant, and weighed again (W3). The fat content (FC, Equation (1)) was calculated (% fat) according to the device manufacturer using the following formula:

FC = [(W2 − W3)/W1] × 100(1)

### 2.4. Calculation of Bioconversion Performance

The weights of diets, total larval biomass (fresh larval weight), and residue, combined with dry matter analyses, allow for estimates of how efficiently larvae converted the experimental diets into larval biomass or how efficiently larvae reduced it. The bioconversion efficiency (BE), bioconversion efficiency corrected for residue (BER), and waste reduction rate (WRR) were calculated. Conversion efficiencies were expressed on a dry matter basis. The BE (%) was estimated with Equation (2). The residue (R) at the end of the experiment, consisting of the diet, exuvia, and excreta, was quantified (in g of dry matter) and used to correct the amount of diet provided based on the Equation (3). The outcome of this formula expresses the BER (%). The formulas used were designed by Bosch, et al. [24]. The waste reduction rate [45] was estimated based on the amount of diet provided during the experiment and the residue obtained at the end of the experiment (in %) (WRR, Equation (4)).

BE = ((L_end_ − L_start_)/D) × 100 (2)

BER = ((L_end_ − L_start_)/(D − R)) × 100 (3)

WRR = ((D − R)/D) × 100(4)

where L_start_ is the average dry matter of the input larvae (g), L_end_ represents the average dry matter of the output larvae (g), D is the average dry matter of the feed (g), and R refers to the average dry matter of the residues from bioconversion (g).

### 2.5. Quantification of Selected Groups of Microorganisms (Microbial Counts)

All larval samples, taken before and after the experiment, were subjected to microbiological analyses aimed at the quantitative detection of the following indicators: total aerobic counts (TACs), Enterobacteriaceae (EB), aerobic bacterial endospores (ABE), yeast (Y), and microscopic filamentous fungi (MFF). Microbial analyses were performed via colony count, according to the relevant ISO standards (4833-1 [46]; 21528-1 [47]; 21527-1 [48]). TACs were analyzed using Plate Count Agar (PCA, Biokar Diagnostics, Beauvais, France) and cultivated at 30 °C for 72 h. EB were determined on Violet Red Bile Glucose agar (VRBG, Biokar Diagnostics, Beauvais, France) after incubation at 37 °C for 24 h. ABE detection was carried out on Plate Count Agar at 37 °C for 48 h after subjecting the basic dilutions of the samples to heat shock (80 °C for 10 min followed by rapid cooling) to eliminate vegetative forms of microorganisms. Y and MFF were incubated on Dichloran Rose Bengal Chloramphenicol agar (DRBC, Biokar Diagnostics, Beauvais, France) at 25 °C for 7 days, and the calculation was performed separately for each group of microscopic fungi.

### 2.6. Microbial Communities of BSFL (DNA Analysis)

From each variant of the experiment, 12 randomly chosen larvae (3 pieces from each container) were subjected to a metabarcoding analysis assessing their microbiome. To monitor the changes caused by different diets, 12 pieces of larvae collected prior to the experiment were also analyzed.

DNA from whole larvae was extracted using the “Gene Jet plant DNA” kit (Thermo Fisher Scientific, Waltham, MA, USA). One whole larva was placed in a microtube and crushed using 4 steel balls in a Beadbug homogenizer (Benchmark Scienfitic, Sayreville, NJ, USA). General bacterial primers 515F and 806R [49] enhanced by an 8 bp identification sequence (barcode) were used for amplification of the V4 section of the bacterial 16S rRNA gene. For analysis of fungal communities, primers gITS7 and ITS4 were used for amplification of the ITS2 region [50]. New England Biolab Q5 polymerase (New England Biolabs, Ipswich, MA, USA) was used for PCR amplification according to manufacturer recommendations. PCR products were purified using an AMPure XP (Beckman Coulter, Brea, CA, USA), quantified by Qubit (Invitrogen/Thermo Fisher Scientific, Waltham, CA, USA), and pooled together in an equimolar ratio. Illumina adapters were ligated using the TruSeq LT PCR free kit (Illumina, San Diego, CA, USA). The library was quantified by qPCR using a Kapa library quantification kit for Illumina (Roche, Basel, Switzerland). The MiSeq Reagent Kit v3 (600-cycle, Illumina, San Diego, CA, USA) was used for sequencing.

Acquired sequencing data were processed in the SEED environment ver. 2.1.4 [51]. Forward and reverse reads were joined with a minimum 50 base overlap. Sequences with quality < Q30 were removed from further analysis. Samples were demultiplexed, and primers were removed. Chimeras were detected de novo by Vsearch ver. 2.1.5 [52]. Chimera-free sequences were clustered into operational taxonomic units (OTUs) using Vsearch at a similarity level of 97%. The most abundant sequence from each cluster (OTU) was found and identified using RDP classifier ver. 2.13 [53].

### 2.7. Data Analysis

The data collected were analyzed using relevant statistical methods to obtain meaningful conclusions and insights. Data about the weight of larvae and their fat and protein content were analyzed by ANOVA in the R statistical environment (R software, version 4.2.2) [54].

For analysis of microbial communities, NonMetric Mulidimensional Scaling and PERMANOVA (Adonis) hypothesis testing was conducted using the package Vegan ver. 2.6-4 [55] in R. Heatmaps were constructed using Heatmap 3 ver. 1.1.9 in R with the complete clustering method based on Bray–Curtis distances. The Shannon diversity index, Pielou evenness, and Chao1 diversity indices were calculated using SEED2 after rarefaction to the lowest sequence count per sample and compared by ANOVA. DESeq2 ver. 1.46.0 [56] was used for differential abundance between the variants on all taxonomic levels.

Multiple correlation analysis between the larvae properties, bioconversion indices, and abundance of bacterial and fungal classes was conducted using Spearman rank correlations and visualized by the Igraph ver. 2.0.3 [57] library in R.

## 3. Results

### 3.1. Food Diet Characteristics and Bioconversion Performance

The measured moisture content (MC) and water activity (a_w_) of individual diet variants are presented in Table 1. The difference in MC and a_w_ between the open storage-tempered variant and the other variants points to the influence of storage conditions. Open storage at room temperature caused a higher moisture content, but at the same time a slightly lower water activity, which may be the result of water evaporation at higher temperatures. Refrigerated closed storage (CS-C) appears to reduce evaporation but still creates an environment with increased water availability for microbial processes.

The bioconversion indices (Table 2) in all three treatments were only minimally different. The differences were evident in the dry matter of the remaining frass. Frass from waste stored at room temperature had a demonstrably lower dry matter content than the other two treatments. The bioconversion efficiency, bioconversion efficiency corrected for residue dry matter content, and waste reduction rate were demonstrably lowest in the OS-T variant. In contrast, the chilled variant had the highest waste reduction.

### 3.2. The Impact of Different Waste Storage Approaches on the Content of Proteins and Lipids in BSFL

The effects of different food waste storage methods on the biometric and nutritional variables of larvae are shown in Table 3. During feeding, the larval weight increased 2.9–3.7 times. The greatest increase in larval weight was recorded in the case of the feeding spoiled waste stored in closed containers at refrigerator temperatures (CS-C). There was no significant difference between larvae fed with the fresh prepared diet and larvae fed with the diet stored at room temperature. However, we also found significant differences in the dry matter content of the larval biomass. The calculated dry weight of single larvae was significantly higher in the CS-C variant while the room temperature-stored variant (OS-T) achieved the lowest values. The dry weight can be considered as the “end product” or an outcome of the larvae’s bioconversion of waste food. The higher the dry weight, the more efficiently the larvae processed the waste. The ash content after experimental feeding was also affected by the diet type; in comparison to input larvae, it significantly decreased in the cold-stored variant while it increased in the room-temperature variant.

The crucial nutritional factors in edible insects are their protein and fat contents. In this study, both parameters changed significantly according to the waste storage method. Prior to the experiment, larvae fed with conventional chicken feed had the highest proportion (%) of protein content while having the lowest proportion of fat. After experimental feeding with kitchen waste, protein content based on the dry matter of larvae had a decreasing trend in the order CS-C > FD > OS-T (with an average value per single larvae of 14.11 mg > 12.45 mg > 9.70 mg, respectively). In contrast, the larvae of the OS-T variant had the highest ratio of fat content, followed by the CS-C and FD variants.

### 3.3. Quantitative Determinations of Microbial Groups

The microbial analysis of the *H. illucens* larvae utilized in the bioconversion process was conducted to assess the presence and quantity of important microbial groups. Table 4 provides insight into the microbial load and potential contaminants in the samples.

Total aerobic counts in larvae before the experiment exhibited an average of 6.76 log CFU/g. This general indicator of the microbial load significantly changed after bioconversion processes only in larvae reared with food waste stored at room temperature. The number of sporulating aerobic bacteria was not altered by feeding on various diets. Enterobacteriaceae, one of the most important groups in health safety considerations, rose in all variants, and it was significantly affected by diet. The OS-T variant larvae showed the most significant rise. The most prominent effect of storage was found in the case of microscopic filamentous fungi. Visually moldy diets also showed remarkably high counts of fungi in the BSFL. Yeast counts rose most significantly in the control—fresh diet—as well as in the variant stored at room temperature. However, the increase in the room temperature-stored diet was smaller than in fresh diet.

### 3.4. Bacterial Diversity

**Figure 1:**
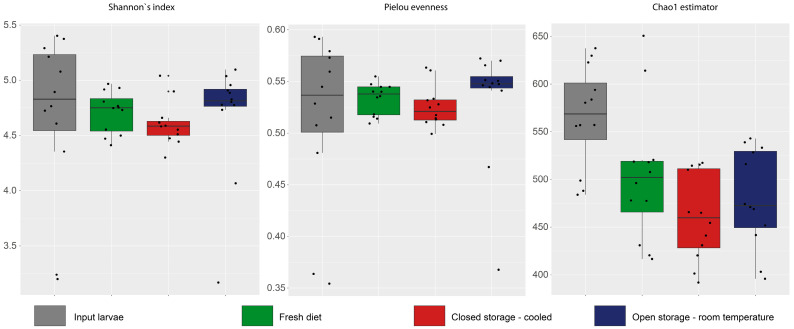
Box and whisker plots of alpha-diversity indices of bacterial community in BSFL reared on diet stored in different conditions.

In total, 1,681,452 obtained sequences of 16S rRNA gene were clustered into 5486 OTUs. Bacterial alpha diversity was not affected by diet storage. There were no differences for the Shannon diversity index or Pielou evenness between the larval groups; although, a significant higher ChaO1 diversity estimator was reported in larvae before experimental feeding (Figure 1).

**Figure 2:**
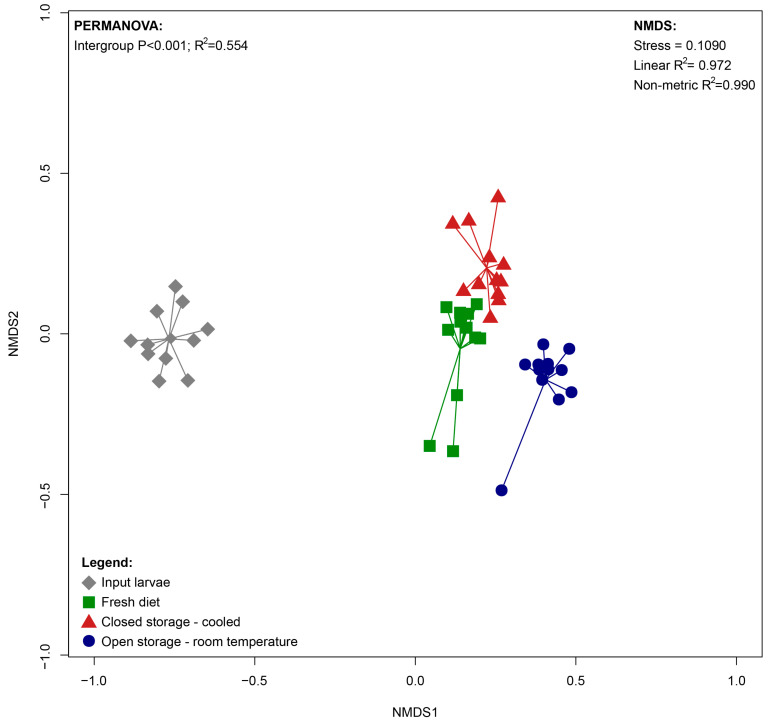
NMDS (Non-Metric Multidimensional Scaling) scatterplot illustrating differences between bacterial community composition of BSFL reared on diet stored in different conditions.

Analysis of Beta diversity (Figure 2) showed a significant shift in the community during experimental feeding. The diet storage method also affected the community (Supplementary Table S1). The differences between the FD and CS-C conditions were less significant (*p* = 0.002; R^2^ = 0.14) in comparison to OS-T (*p* = 0.001; R^2^ = 0.29).

**Figure 3:**
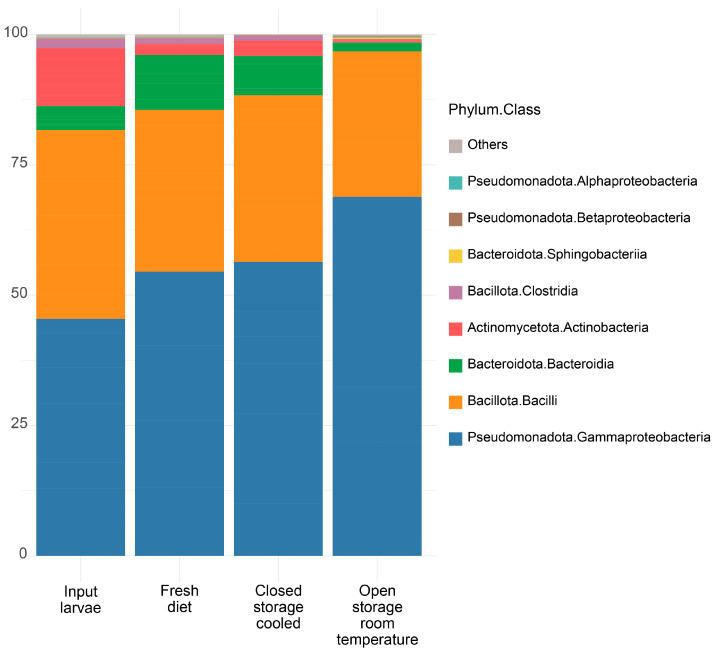
Average bacterial community composition at class level in BSFL reared on diet stored in different conditions.

More than 99% of the community was made up of four classes of bacteria (Figure 3). During experimental larvae feeding, Actinobacteria decreased from 11% to 0.5–2.9% (all *p* < 0.05). Gammaproteobacteria were significantly more (69% vs. 54%; *p* < 0.001) and Bacteroidia were less (1.8 vs. 10.6%; *p* < 0.001) abundant in the OS-T variant compared to the FD condition. The detailed differential abundance analysis is reported in Supplementary Table S2.

**Figure 4:**
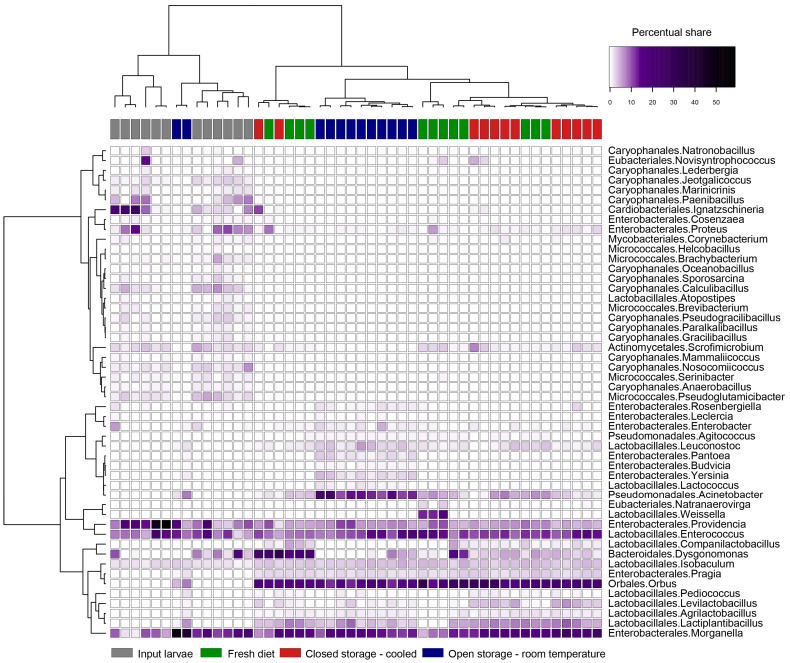
Heatmap of 44 most abundant bacterial genera detected in BSFL reared on diet stored in different conditions.

Figure 4 shows the results of the bacterial profile investigation on the genera level. The most common bacterial genera identified across all variants were *Morganella*, *Enterococcus*, and *Providencia*. The microbiomes of input larvae contained several genera like *Proteus*, *Paenibacillus*, and *Ignatzschineria* which lowered in abundance during experimental feeding. However, *Orbus, Dysgonomonas*, or *Lactiplantibacilllus* significantly rose in abundance. Sequences of *Acinetobacter, Leuconostoc*, *Pantoea*, or *Yersinia* appeared mainly in the OS-T variant. Only a few genera like *Levilactobacillus* and *Ignatzschineria* were significantly more common in the CS-C variant than in the fresh diet.

### 3.5. Fungal Diversity

**Figure 5:**
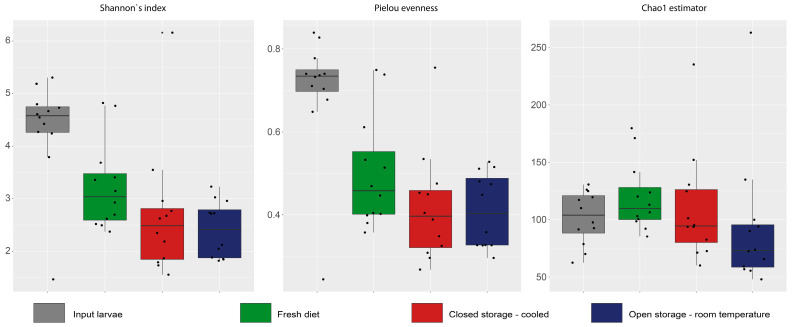
Box and whisker plots of alpha-diversity indices of fungal community in BSFL reared on diet stored in different conditions.

In total, 681,453 sequences clustered into 711 OTUs were obtained for the ITS region. Analysis of fungal diversity showed a significantly higher Shannon index and Pielou evenness result for input larvae (Figure 5). Diversity in the OS-T variant was lower than for the fresh prepared diet. The Chao1 index result was not significantly different.

**Figure 6:**
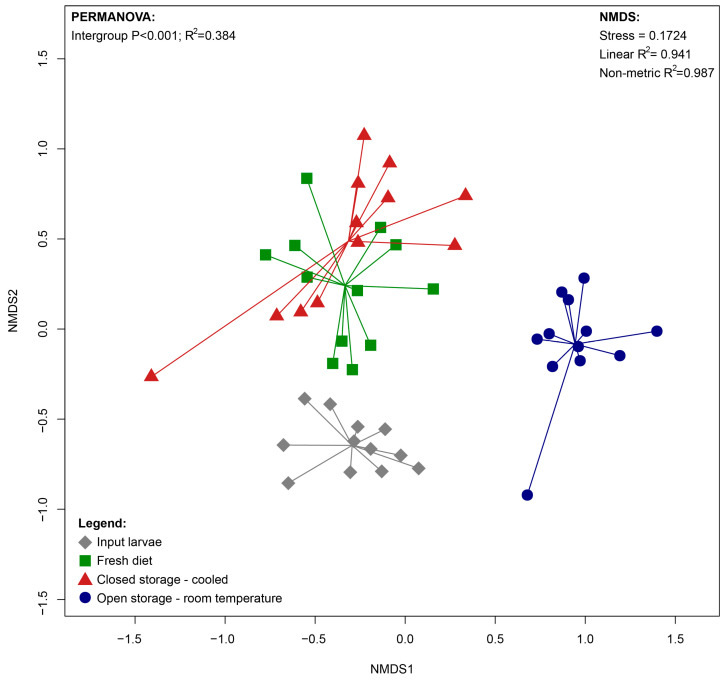
NMDS (Non-Metric Multidimensional Scaling) scatterplot illustrating differences between fungal community composition of BSFL reared on diet stored in different conditions.

Analysis of Beta diversity (Figure 6) showed a significant shift in the community during feeding (Supplementary Table S3). The diet storage method also affected the community. The differences between the FD and CS-C conditions were not significant in contrast to the open storage variant (*p* = 0.001; R^2^ = 0.47).

**Figure 7:**
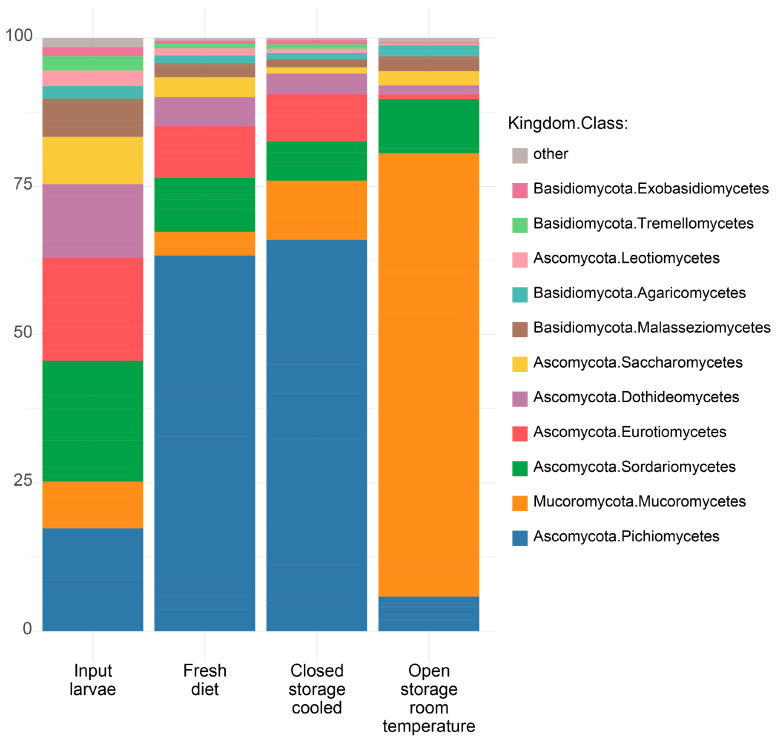
Average fungal community composition at class level in BSFL reared on diet stored in different conditions.

Changes in evenness are apparent in the distribution of fungal classes (Figure 7). Input larvae show a more balanced distribution across multiple classes, with no single class overwhelmingly dominant while the FD and CS-C conditions support the strong development of *Pichiomycetes*, and the OS-T condition led to the overgrowth of *Mucoromycetes*.

**Figure 8:**
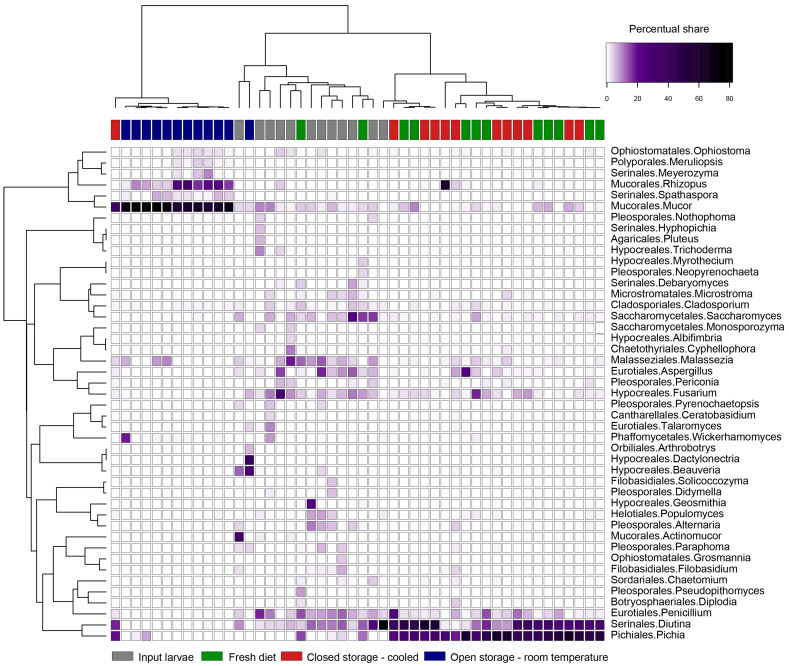
Heatmap of 44 most abundant fungal genera detected in BSFL reared on diet stored in different conditions.

The distribution of fungal genera, which is summarized in Figure 8, was highly dependent on the particular variant. In the mycobiome of the input larvae, genera differed in abundance among the samples (Supplementary Table S4). Dominant genera often included *Penicillium*, *Malassezia*, *Fusarium*, and others, depending on the particular sample. Yeast from the genera *Pichia* and *Diutia* were the most common in larvae fed with the FD and CS-C despite the fact that they were poorly abundant in the OS-T variant and input larvae. On the other side, conditions in open stored containers suppressed not only the growth of *Pichia* and *Diutia* but also some other genera which were common in input larvae. An open stored diet offers optimal conditions for the development of micromycetes like *Mucor* and *Rhizopus*.

### 3.6. Correlations Between Bioconversion and the Larval Microbiome

**Figure 9:**
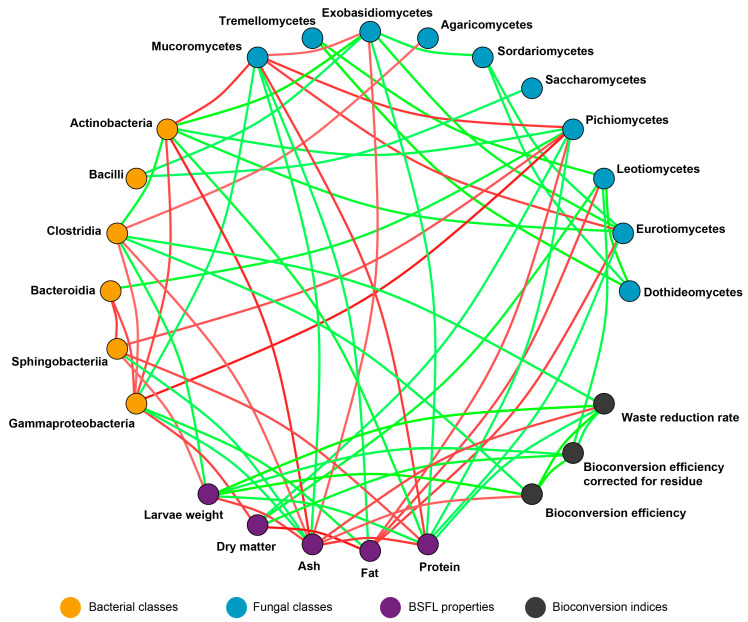
Correlation network diagram showing relationships between BSFL properties, bioconversion indices, and abundance of bacterial and fungal classes. Only significant correlations (*p* < 0.05) are visualized. Green lines represent positive correlations between variables and red lines negative correlations.

To explore the relationships between the bioconversion efficiency of BSFL, their nutritional parameters, and microbial community dynamics, a multiple correlation analysis was conducted. Spearman rank correlations highlighted significant associations, which are visualized in Figure 9. These findings provide an integrated perspective, tying process performance with microbial dynamics. Analysis between larvae properties, bioconversion indices, and abundance of bacterial and fungal classes showed 39 positive and 26 negative correlations. Significant correlations were found between microbiological profiles of BSFL and bioconversion efficiency, as well as between the nutritional quality of larvae and their microbial composition.

The only class that showed a positive correlation with bioconversion efficiency (BE) and waste reduction rate (WRR) was Clostridia, which was also positively linked with larval weight. In contrast, the BSFL weight had a negative association with the presence of bacteria belonging to the Sphingobacteriia class. The abundance of members of this class was also inversely connected with the content of protein in the larvae. Regardless, microbial diversity had a significant relationship with the larvae’s protein content, which is an important indicator of their utility. Higher content was positively linked to Eurotiomycetes, Pichiomycetes, Exobasidiomycetes, and Actinobacteria but was reduced in the presence of spoilage-associated fungi of the Mucoromycetes class and bacteria from the Sphingobacteriia class.

## 4. Discussion

Our study demonstrates how different food waste storage methods significantly influence the bioconversion process by *Hermetia illucens* larvae (BSFL). Specifically, the storage conditions affected waste reduction efficiency, feed conversion rates, nutritional content of the larvae, and their potential safety in terms of the microbial profile of the resulting larval biomass.

Refrigerated, closed storage of food waste in the CS-C variant resulted in the highest waste reduction (91.0%) and bioconversion efficiency (30.2%), while open, room-temperature storage (OS-T) showed reduced bioconversion rates. This highlights the critical role of pre-consumption substrate quality in optimizing the biodegradation process and improving larval productivity.

The effect of FW storage on nutritional content was equally profound. The protein content of larvae in our study followed the trend CS-C > FD > OS-T, with the CS-C variant producing larvae with significantly higher protein accumulation. This suggests that refrigerated storage preserved protein precursors in the waste, leading to better nutritional outcomes. Protein precursors, mainly amino acids, are molecules that contribute to the synthesis of proteins [58]. These precursors are critical for larval growth as they provide the building blocks for de novo protein synthesis. In the context of *Hermetia illucens* larvae, substrates rich in digestible amino acids and peptides may promote efficient protein assimilation and deposition into larval biomass [59]. However, as the protein content we have reported is a relative percentage, it should be noted that it may be affected by the accumulation of other components such as lipids or chitin. An increase in these components may, of course, proportionally reduce the measured percentage of protein, although there is no absolute reduction in protein synthesis. Future studies should aim to analyze the absolute protein content or consider the total nitrogen balance to better account for these variations. The OS-T variant, conversely, supported higher fat accumulation, likely due to microbially mediated degradation of complex organic matter into simpler lipids, fatty acids, and amino acids [26]. These results highlight the importance of tailoring FW storage conditions to optimize larval biomass composition depending on its intended use (e.g., protein feed vs. lipid-rich bioresources). This aligns with previous findings [25], where substrates with extensive microbial activity yielded lower protein content in BSFL. In this research, it was shown that the larvae in the variant with the longest storage time and, consequently, the highest microbial decomposition had demonstrably higher fat content. The larvae’s protein content was higher when the feed was stored for a shorter time and, therefore, was less microbiologically degraded.

Additionally, our findings underline that FW storage conditions directly influence the microbial load and composition of BSFL. The temperature during storage or transport is an essential element that affects the rate of development of microorganisms in processed waste. The OS-T variant resulted in the highest microbial counts, including Enterobacteriaceae and molds, which are indicators of reduced microbiological safety. This is consistent with studies showing that open and warm storage of FW promotes the growth of potentially harmful microorganisms [34,37]. Our findings were in accordance with the numbers of microorganisms summarized by Garofalo, et al. [30]. According to this study, fresh edible insects generally contain high microbial loads, including total mesophilic aerobes (3.6–9.4 log CFU/g), Enterobacteriaceae (4.2–7.8 log CFU/g), bacterial endospores or spore-forming bacteria (0.5–5.8 log CFU/g), and yeasts and molds (3.4–7.2 log CFU/g). The authors also note that processed edible insects generally showed lower microbial counts than fresh insects, indicating the effectiveness of some treatments in reducing microbial contamination of these novel potential foods.

Microbial degradation during the different storage management methods for food waste can have a dual role in the process of bioconversion by BSFL. On the one hand, microbial activity can initiate the breakdown of complex organic compounds, rendering the substrate more accessible and palatable to the larvae [19]. On the other hand, excessive microbial degradation can lead to nutrient loss or the accumulation of metabolites, such as organic acids or ethanol, that may inhibit larval growth or feed conversion efficiency [36]. Based on the bioconversion indices (bioconversion efficiency and waste reduction rate) obtained in our study, waste stored at lower temperatures (CS-C) appeared to be the most suitable substrate, with the indices decreasing in the order FD > OS-T. These findings suggest that microbial development during cold storage of waste could provide a balance between pre-fermentation of the substrate and preservation of its nutritional value. This nuanced understanding highlights the need to consider not only the storage conditions themselves but also their downstream effects on the bioconversion process, as microbial load and diversity appears to play a significant role in determining the substrate’s suitability for BSFL. In our research, we focused on the total larval microbiome, including both the gut microbiome and microorganisms of the external parts of the insect. The BSFL microbiome was dominated by genera such as *Morganella*, *Enterococcus*, and *Providencia*, which are abundantly associated with BSFL reared on various organic substrates [25,32,33,39,60,61]. These genera play a role in nutrient breakdown and larval digestion but can also include health-threatening species. According to Raimondi et al. [38], with respect to microbiological risk assessment, attention should be paid mainly to abundant genera such as spore-forming *Bacillus* or *Providencia*, *Morganella,* and *Proteus*, which were also found in our study. They encompass species described as opportunistic pathogens, bearing drug resistances or causing severe morbidity. Heat treatment of waste can be used to eliminate them, but it is more economically demanding. According to Van Looveren et al. [42], the use of a 60 °C treatment for 10 min can reduce contamination with Enterobacteriaceae or *Salmonella* spp. to less than 1 log. Waste treated in this way can be stored for at least 2 days at 4 °C without increased microbiological hazard.

*Orbus*, the most common bacterial genera detected in larvae after experimental feeding, is a well-known insect gut symbiotic bacteria, strongly correlated with the metabolism of non-protein amino acids [32]. Compared to other studies [25,62], we found a lower abundance of Clostridiaceae. This was probably caused by aerobic feed storage, which limited the growth of the Clostridiaceae family species that are strictly anaerobic. Lactic acid bacteria such as *Lactiplantibacillus, Leuconostoc,* or *Weisella* were highly abundant as previously reported in larval gut or feed samples [25,63]. On the one hand, the overlap of microbial profiles observed in this and other studies [32,39] confirms the consistent role of the BSFL microbiome in bioconversion. On the other hand, however, the presence of opportunistic pathogens in some conditions underlines the need for further research on how husbandry conditions, including FW storage, affect the balance between beneficial and harmful microorganisms.

In the area of microscopic fungi, the most significant occurrence of yeasts was mainly *Pichia* or *Diutia*, which were dominant in both the fresh and cooled food waste diet. In the case of waste stored at room temperature, there was a rapid development of filamentous fungi such as *Mucor* and *Rhizopus*, associated with a decrease in the proportion of the aforementioned yeasts. Our data show a much higher dependence of the fungal part of the microbiome on the substrate and external factors compared to the bacterial component. The presence of detected fungi in the OS-T variant highly reflects the substrate’s condition, further emphasizing the need for controlled FW storage. Previous studies have also documented a similarly strongly stochastic composition [33,40]. At the same time, these authors highlight the relationship of yeasts from the genus *Pichia* to the occurrence of potentially pathogenic bacteria. The antibacterial proteins and organic acids produced by these yeasts can suppress the development of pathogens such as *E. coli*, *Enterococcus* spp., or *Klebsiella* spp. In our case, the absence of *Pichia* spp. in the OS-T variant correlated with the presence of *Acinetobacter* spp. or several Enterobacteriales such as *Yersinia* spp. and *Pantoea* spp.

A comprehensive look at the results of our study shows many important relationships between bioconversion performance and larval microbiome, as well as between the microbial and nutritional quality of the BSFL. Among the bacterial classes, Clostridia exhibited a positive correlation with the bioconversion efficiency (BE), waste reduction rate (WRR), and larval weight. This aligns with research highlighting the role of such bacteria in degradation processes, where they convert complex organic substrates into short-chain fatty acids and other metabolites that support larval growth [19,36]. Conversely, Sphingobacteriia showed a negative association with larval weight and protein content, suggesting potential competitive or inhibitory interactions within the microbiome. These bacteria are often associated with nutrient competition and the breakdown of proteins into less bioavailable forms, which can reduce the efficiency of nutrient utilization by larvae [33]. This highlights the need for strategies to limit Sphingobacteriia abundance during substrate preparation or storage to improve larval biomass quality.

In the fungal microbiome, Pichiomycetes (dominated by *Pichia* species) correlated positively with protein content in larvae. Together with the aforementioned antagonistic relationships with harmful bacteria, these yeasts appear to be relatively beneficial. This dual role in nutrient enhancement and microbial safety makes them a valuable component of the larval microbiome. These insights align with previous studies demonstrating the potential of microbiome management to optimize bioconversion systems [25]. Strategies such as selective inoculation with beneficial microbes (e.g., *Pichia*) or the addition of prebiotic compounds to substrates could further enhance larval growth and nutrient accumulation [39]. On the other hand, spoilage-associated fungi like Mucoromycetes were prevalent in diets stored at room temperature and negatively associated with protein content, likely due to the degradation of protein precursors and the accumulation of less favorable metabolites [40]. These findings emphasize the importance of controlled storage conditions to limit fungal overgrowth and preserve substrate quality.

Our results also highlight the potential of microbiome-driven strategies to enhance the sustainability and efficiency of BSFL-based bioconversion systems. In order to maximize the quantity and quality of larval biomass, this study establishes a basis not only for the development of targeted interventions through the management of waste handling, but also through the identification of microbial taxa that either enhance or limit process performance. To confirm these associations and determine causal relationships, future studies should focus on experimental modification of microbial communities to ensure reliable and scalable applications under different substrate conditions.

## 5. Conclusions

The results demonstrate that bioconversion by BSFL can effectively process FW regardless of its storage method, but the quality and safety of the resulting *H. illucens* larval biomass are highly dependent on substrate conditions. While FW in all three variants was converted into biomass, the nutritional and microbiological outcomes varied significantly. According to our results, to achieve the best outcomes in terms of waste reduction, bioconversion efficiency, and the nutritional and microbial quality of larval biomass, food waste should be stored in refrigerated, closed conditions. Storage at a temperature of 5–8 °C (compared to a fresh diet and a diet stored at room temperature), through changes in the microbiological properties of the substrate, led to the improvement of the nutritional parameters of the larvae and bioconversion indices and minimized the spread of harmful microorganisms. In contrast, storage in open, room-temperature conditions leads to reduced bioconversion performance, higher microbial loads, and unfavorable microbial profiles, including spoilage fungi and potential bacterial pathogens. Controlled storage conditions (particularly refrigeration), therefore, not only ensure higher protein content and optimal larval growth but also suppress undesirable microbial activity that could compromise the safety and quality of the larvae.

## Figures and Tables

**Table 1:** Characteristics of feed variants used in BSFL feeding process for the purpose of food waste biodegradation in terms of moisture content (MC) and water activity (a_w_). Values are the mean ± standard deviation.

Variant	MC [%]	a_w_
FD	70.07 ± 1.18	0.818 ± 0.005
CS-C	69.87 ± 2.20	0.874 ± 0.010
OS-T	73.69 ± 1.02	0.811 ± 0.005

FD—freshly prepared feed; CS-C—feed naturally contaminated with microorganisms and stored in closed containers for 5 days at refrigerated temperature; OS-T—feed naturally contaminated with microorganisms and stored in opened containers for 5 days at room temperature.

**Table 2:** Efficiency of BSFL bioconversion of food waste stored in different ways. Values are the mean ± standard deviation (*n* = 4). Values accompanied by the same letter (within columns) are not significantly different, while values with different letters indicate significant differences (ANOVA, LSD test, *α* = 0.05).

Variant	BE [%]	BER [%]	WRR [%]	DM_R_ [%]
FD	27.4 ± 2.8 ^a^	33.6 ± 1.8 ^a^	81.3 ± 4.4 ^a^	66.0 ± 1.5 ^b^
CS-C	30.2 ± 2.8 ^a^	33.2 ± 1.8 ^a^	91.0 ± 5.5 ^b^	66.5 ± 1.7 ^b^
OS-T	23.5 ± 5.0 ^a^	30.1 ± 3.4 ^a^	77.6 ± 7.5 ^a^	57.4 ± 1.5 ^a^

BE—bioconversion efficiency; BER—bioconversion efficiency corrected for residue; WRR—waste reduction rate; DM_R_—dry matter of the residues; FD—larvae fed with freshly prepared feed; CS-C—larvae fed with feed naturally contaminated with microorganisms and stored in closed containers for 5 days at refrigerated temperatures; OS-T—larvae fed with feed naturally contaminated with microorganisms and stored in opened containers for 5 days at room temperature.

**Table 3:** Proximate composition analysis of BSFL samples (* based on dry matter). Values are the mean ± standard deviation (*n* = 4). Values accompanied by the same letter (within columns) are not significantly different, while values with different letters indicate significant differences (ANOVA, LSD test, *α* = 0.05).

Variant	FW [mg/1 larva]	DW [mg/1 larva]	DM [%]	AC * [%]	PC * [%]	FC * [%]
IL	31.3 ± 1.7 ^a^	5.92 ± 0.32 ^a^	18.94 ± 0.31 ^a^	6.97 ± 0.2 ^a^	37.5 ± 0.19 ^d^	25.1 ± 0.13 ^a^
FD	100.0 ± 8.1 ^b^	35.11 ± 2.97 ^c^	35.1 ± 0.29 ^d^	7.02 ± 0.13 ^b^	35.5 ± 0.68 ^b^	25.81 ± 0.18 ^b^
CS-C	115.3 ± 7.4 ^c^	38.34 ± 2.99 ^c^	33.25 ± 1.01 ^c^	6.58 ± 0.11 ^b^	36.83 ± 0.22 ^c^	26.83 ± 0.12 ^c^
OS-T	89.5 ± 12.5 ^b^	27.94 ± 4.66 ^b^	31.14 ± 0.84 ^b^	7.44 ± 0.37 ^c^	34.71 ± 0.43 ^a^	34.02 ± 0.12 ^d^

FW—fresh weight; DW—dry weight; DM—dry matter; AC—ash content; PC—protein content; FC—fat content; IL—input larvae; FD—larvae fed with freshly prepared feed; CS-C—larvae fed with feed naturally contaminated with microorganisms and stored in closed containers for 5 days at refrigerated temperature; OS-T—larvae fed with feed naturally contaminated with microorganisms and stored in opened containers for 5 days at room temperature.

**Table 4:** Counts of cultivable microorganisms in BSFL before (IL = input larvae) and after bioconversion process (FD, OS-T, and CS-C). Values are the means of log_10_ CFU/g counts ± standard deviations (*n* = 4). Values accompanied by the same letter (within columns) are not significantly different, while values with different letters indicate significant differences (ANOVA, LSD test, *α* = 0.05).

Variant	TAC	ABE	EB	MFF	Y
IL	6.76 ± 0.41 ^a^	5.54 ± 1.03 ^a^	5.66 ± 0.26 ^a^	3.64 ± 0.07 ^b^	3.94 ± 0.08 ^a^
FD	6.74 ± 0.21 ^a^	4.93 ± 0.10 ^a^	6.94 ± 0.38 ^b^	1.27 ± 0.38 ^a^	5.78 ± 0.23 ^c^
CS-C	6.70 ± 0.20 ^a^	5.16 ± 0.08 ^a^	6.92 ± 0.57 ^b^	1.40 ± 0.49 ^a^	4.46 ± 0.56 ^a^
OS-T	7.28 ± 0.06 ^b^	5.83 ± 0.30 ^a^	7.66 ± 0.23 ^c^	5.33 ± 0.42 ^c^	5.05 ± 0.36 ^b^

TAC—total aerobic count; ABE—aerobic bacterial endospores; EB—Enterobacteriaceae; MFF—microscopic filamentous fungi; Y—yeast; IL—input larvae; FD—larvae fed with freshly prepared feed; CS-C—larvae fed with feed naturally contaminated with microorganisms and stored in closed containers for 5 days at refrigerated temperature; OS-T—larvae fed with feed naturally contaminated with microorganisms and stored in opened containers for 5 days at room temperature.

## Data Availability

Sequencing data produced in this study are available in the National Centre for Biotechnology Information’s (NCBI) Sequence Read Archive (SRA) repository, under BioProject ID PRJNA1182311.

## Supplementary Materials

The following supporting information can be downloaded at: https://www.mdpi.com/article/10.3390/insects16010087/s1, Table S1: Pairwise comparison of bacterial community difference (PERMANOVA); Table S2: Differential abundance analysis of Bacteria (only members with more than 1% in any variant are listed); Table S3: Pairwise comparison of fungal community difference (PERMANOVA); Table S4: Differential abundance analysis of Fungi (only members with more than 1% in any variant are listed). The data presented in this study are available on request from the corresponding author.
