# Supplementary material for: Hermetia illucens in the Process of Kitchen Waste Biodegradation: The Effect of Different Approaches to Waste Storage on the Microbiological Profile and Nutritional Parameters of the Larvae
Source: Insects. 2025 Jan 16;16(1):87. doi: 10.3390/insects16010087 (PMC11765827; doi:10.3390/insects16010087)
Supplement: Supplementary file 1 [file insects-16-00087-s001.zip › insects-3345497-supplementary.pdf]

Table S1: Pairwise comparion of bacterial community difference (PERMANOVA)

p values

		IL	FD	CS-C	OS-T
	IL	NA	0.001	0.001	0.001
	FD	0.4954187	NA	0.002	0.001
	CS-C	0.5381897	0.1402439	NA	0.001
R2 values	OS-T	0.5307981	0.2904501	0.3499189	NA

Legend:

IL – input larvae

FD – larvae fed by freshly prepared feed

CS-C – larvae fed by feed naturally contaminated with microorganisms and stored in closed containers for 5 days at refrigerated temperature

OS-T – larvae fed by feed naturally contaminated with microorganisms and stored in opened containers for 5 days at room temperature

NA - Not Applicable

Table S2: Differential abundance analysis of Bacteria (only members with more than 1% in any variant are listed)

Phylogenetic group							Mean occurence in variant				DeSeq2 Log2fold ratio (*p=<0.05;**p=<0.01)			
domain	phylum	class	order	family	genus	OTU	IL	FD	CS-C	OS-T	IL vs FD	OS-T vs FD	OS-T vsCS-C	FD vsCS-C
Bacteria	Actinomycetota						11.082	0.528	2.146	2.946	-2.052**	1.341**	1.695**	<0.001
Bacteria	Actinomycetota	Actinobacteria					11.077	0.520	2.120	2.944	-2.129**	0.816**	1.131**	<0.001
Bacteria	Actinomycetota	Actinobacteria	Actinomycetales				2.974	0.228	1.357	2.240	<0.001	2.062**	2.795**	0.778*
Bacteria	Actinomycetota	Actinobacteria	Actinomycetales	Actinomycetaceae			2.974	0.228	1.357	2.240	-0.976**	2.529**	3.285**	0.875**
Bacteria	Actinomycetota	Actinobacteria	Actinomycetales	Actinomycetaceae	Scrofimicrobium		2.837	0.226	1.352	2.238	-0.929**	2.306**	2.773**	<0.001*
Bacteria	Actinomycetota	Actinobacteria	Actinomycetales	Actinomycetaceae	Scrofimicrobium	CL00017	1.131	0.122	0.919	1.358	<0.001	3.154**	3.089**	<0.001
Bacteria	Actinomycetota	Actinobacteria	Actinomycetales	Actinomycetaceae	Scrofimicrobium	CL00038	1.035	0.008	0.042	0.012	-4.662**	2.331**	<0.001	<0.001
Bacteria	Actinomycetota	Actinobacteria	Micrococcales				7.218	0.184	0.436	0.272	-3.219**	<0.001	0.173	<0.001
Bacteria	Actinomycetota	Actinobacteria	Micrococcales	Beutenbergiaceae			1.408	0.019	0.291	0.057	-2.139**	3.952**	1.655**	-1.77**
Bacteria	Actinomycetota	Actinobacteria	Micrococcales	Beutenbergiaceae	Serinibacter		1.094	0.010	0.206	0.038	-2.335**	4.347**	1.398**	<0.001**
Bacteria	Actinomycetota	Actinobacteria	Micrococcales	Brevibacteriaceae			1.139	0.001	0.015	0.012	-6.189**	<0.001*	1.821	<0.001
Bacteria	Actinomycetota	Actinobacteria	Micrococcales	Dermabacteraceae			1.626	0.001	0.018	0.004	-6.496**	<0.001**	0.666	<0.001
Bacteria	Actinomycetota	Actinobacteria	Micrococcales	Dermabacteraceae	Brachybacterium		1.254	0.000	0.012	0.004	<0.001**	<0.001*	<0.001	<0.001
Bacteria	Actinomycetota	Actinobacteria	Micrococcales	Dermabacteraceae	Brachybacterium	CL00034	1.116	0.000	0.011	0.004	-7.012**	<0.001*	<0.001	<0.001
Bacteria	Actinomycetota	Actinobacteria	Micrococcales	Micrococcaceae			2.931	0.102	0.093	0.112	-4.858**	<0.001	0.105	<0.001
Bacteria	Actinomycetota	Actinobacteria	Micrococcales	Micrococcaceae	Pseudoglutamicibacter		2.701	0.003	0.033	0.003	-6.475**	2.957**	<0.001	<0.001*
Bacteria	Actinomycetota	Actinobacteria	Micrococcales	Micrococcaceae	Pseudoglutamicibacter	CL00025	1.617	0.002	0.019	0.000	-6.55**	<0.001*	<0.001	<0.001
Bacteria	Bacillota						38.056	27.987	32.128	32.974	0.046	<0.001*	-0.611**	<0.001
Bacteria	Bacillota	Bacilli					36.216	27.864	31.008	31.975	<0.001	<0.001**	-1.165**	<0.001
Bacteria	Bacillota	Bacilli	Caryophanales				22.279	0.410	0.279	0.088	-5.435**	-0.739**	-2.516**	-1.302**
Bacteria	Bacillota	Bacilli	Caryophanales	Bacillaceae			10.742	0.305	0.114	0.035	<0.001**	<0.001**	-2.859**	-1.112**
Bacteria	Bacillota	Bacilli	Caryophanales	Bacillaceae	Calculibacillus		2.959	0.002	0.006	0.000	-8.762**	<0.001	<0.001	<0.001
Bacteria	Bacillota	Bacilli	Caryophanales	Bacillaceae	Calculibacillus	CL00018	2.693	0.002	0.005	0.000	-9.274**	<0.001	<0.001	<0.001
Bacteria	Bacillota	Bacilli	Caryophanales	Bacillaceae	Pseudogracilibacillus		1.459	0.001	0.007	0.001	-7.501**	<0.001	<0.001	<0.001
Bacteria	Bacillota	Bacilli	Caryophanales	Caryophanaceae			1.369	0.026	0.005	0.005	-7.565**	<0.001*	-1.756*	<0.001
Bacteria	Bacillota	Bacilli	Caryophanales	Caryophanaceae	Sporosarcina		1.051	0.001	0.001	0.001	-9.982**	<0.001	<0.001	<0.001
Bacteria	Bacillota	Bacilli	Caryophanales	Paenibacillaceae			4.693	0.006	0.022	0.011	-7.464**	<0.001	0.446	<0.001
Bacteria	Bacillota	Bacilli	Caryophanales	Paenibacillaceae	Marinicrinis		1.054	0.001	0.010	0.002	-6.279**	<0.001*	<0.001	<0.001

Bacteria	Bacillota	Bacilli	Caryophanales	Paenibacillaceae	Paenibacillus		3.636	0.005	0.013	0.008	-7.711**	<0.001	<0.001	<0.001
Bacteria	Bacillota	Bacilli	Caryophanales	Paenibacillaceae	Paenibacillus	CL00014	3.504	0.001	0.013	0.006	-8.217**	<0.001**	<0.001	<0.001
Bacteria	Bacillota	Bacilli	Caryophanales	Staphylococcaceae			5.467	0.058	0.133	0.037	-5.22**	<0.001*	-0.619	<0.001**
Bacteria	Bacillota	Bacilli	Caryophanales	Staphylococcaceae	Jeotgalicoccus		1.698	0.003	0.009	0.000	-7.209**	<0.001	<0.001	<0.001
Bacteria	Bacillota	Bacilli	Caryophanales	Staphylococcaceae	Nosocomiicoccus		2.266	0.004	0.017	0.002	-6.881**	<0.001*	<0.001	<0.001
Bacteria	Bacillota	Bacilli	Caryophanales	Staphylococcaceae	Nosocomiicoccus	CL00023	1.632	0.002	0.011	0.002	-7.304**	<0.001*	<0.001	<0.001
Bacteria	Bacillota	Bacilli	Lactobacillales				13.936	27.455	30.729	31.887	2.074**	<0.001	-0.145	<0.001
Bacteria	Bacillota	Bacilli	Lactobacillales	Carnobacteriaceae			2.757	3.640	3.924	3.507	0.529**	<0.001	0.09	<0.001
Bacteria	Bacillota	Bacilli	Lactobacillales	Carnobacteriaceae	Isobaculum		1.969	3.559	3.803	3.417	0.873**	<0.001	<0.001	<0.001
Bacteria	Bacillota	Bacilli	Lactobacillales	Carnobacteriaceae	Isobaculum	CL00021	0.007	0.788	0.901	1.049	6.863**	<0.001	<0.001	<0.001
Bacteria	Bacillota	Bacilli	Lactobacillales	Enterococcaceae			10.796	11.895	11.292	11.350	<0.001	<0.001	0.024	0.227**
Bacteria	Bacillota	Bacilli	Lactobacillales	Enterococcaceae	Enterococcus		10.393	11.607	11.076	11.149	<0.001	<0.001	-0.248	<0.001
Bacteria	Bacillota	Bacilli	Lactobacillales	Enterococcaceae	Enterococcus	CL00003	9.783	10.654	10.159	9.961	<0.001	<0.001	<0.001*	<0.001
Bacteria	Bacillota	Bacilli	Lactobacillales	Lactobacillaceae			0.078	7.850	9.873	15.492	7.117**	<0.001	1.163**	<0.001
Bacteria	Bacillota	Bacilli	Lactobacillales	Lactobacillaceae	Agrilactobacillus		0.021	1.255	1.118	1.518	5.879**	<0.001	<0.001	<0.001
Bacteria	Bacillota	Bacilli	Lactobacillales	Lactobacillaceae	Companilactobacillus		0.012	0.002	2.217	1.265	7.431**	10.169**	9.161**	<0.001
Bacteria	Bacillota	Bacilli	Lactobacillales	Lactobacillaceae	Lactiplantibacillus		0.025	4.347	4.795	6.664	7.69**	<0.001	<0.001	<0.001
Bacteria	Bacillota	Bacilli	Lactobacillales	Lactobacillaceae	Lactiplantibacillus	CL00011	0.010	2.491	2.568	3.572	7.81**	<0.001	<0.001	<0.001
Bacteria	Bacillota	Bacilli	Lactobacillales	Lactobacillaceae	Levilactobacillus		0.007	1.150	0.482	4.228	6.284**	<0.001	1.673**	3.053**
Bacteria	Bacillota	Bacilli	Lactobacillales	Lactobacillaceae	Levilactobacillus	CL00015	0.004	0.859	0.291	3.280	6.108**	<0.001	1.654**	3.415**
Bacteria	Bacillota	Bacilli	Lactobacillales	Leuconostocaceae			0.036	2.988	5.550	1.467	7.105**	<0.001*	-0.796*	-1.143**
Bacteria	Bacillota	Bacilli	Lactobacillales	Leuconostocaceae	Leuconostoc		0.025	2.874	1.912	1.284	6.451**	<0.001	-1.181**	<0.001
Bacteria	Bacillota	Bacilli	Lactobacillales	Leuconostocaceae	Leuconostoc	CL00020	0.012	1.739	0.420	0.274	5.037**	<0.001**	-2.903**	<0.001
Bacteria	Bacillota	Bacilli	Lactobacillales	Leuconostocaceae	Weissella		0.009	0.030	3.473	0.115	7.981**	7.071**	1.608**	-4.218**
Bacteria	Bacillota	Bacilli	Lactobacillales	Leuconostocaceae	Weissella	CL00039	0.002	0.008	1.966	0.026	9.22**	8.201	<0.001	-5.94
Bacteria	Bacillota	Bacilli	Lactobacillales	Streptococcaceae			0.026	1.048	0.069	0.059	1.242**	-3.645**	-3.863**	<0.001
Bacteria	Bacillota	Clostridia					1.837	0.123	1.116	0.997	<0.001	2.26**	1.837**	<0.001
Bacteria	Bacillota	Clostridia	Eubacteriales				1.837	0.123	1.116	0.997	<0.001	2.446**	2.315**	<0.001
Bacteria	Bacillota	Clostridia	Eubacteriales	Lachnospiraceae			1.814	0.114	0.622	0.797	<0.001	1.529*	2.704**	<0.001
Bacteria	Bacillota	Clostridia	Eubacteriales	Lachnospiraceae	Novisyntrophococcus		1.660	0.032	0.405	0.718	<0.001	3.499**	4.216**	<0.001
Bacteria	Bacillota	Clostridia	Eubacteriales	Lachnospiraceae	Novisyntrophococcus	CL00026	1.289	0.010	0.228	0.476	<0.001*	4.402**	5.07**	<0.001
Bacteria	Bacteroidota						5.102	2.227	10.818	7.599	1.441*	1.72**	1.07	<0.001
Bacteria	Bacteroidota	Bacteroidia					4.628	1.751	10.572	7.508	<0.001	1.809**	1.17	<0.001

Bacteria	Bacteroidota	Bacteroidia	Bacteroidales				4.628	1.751	10.572	7.508	2.416**	2.067**	1.746**	<0.001
Bacteria	Bacteroidota	Bacteroidia	Bacteroidales	Dysgonomonadaceae			4.499	1.747	10.517	7.465	<0.001*	2.688**	2.322**	<0.001
Bacteria	Bacteroidota	Bacteroidia	Bacteroidales	Dysgonomonadaceae	Dysgonomonas		4.499	1.747	10.517	7.465	<0.001*	2.642**	1.922*	<0.001
Bacteria	Bacteroidota	Bacteroidia	Bacteroidales	Dysgonomonadaceae	Dysgonomonas	CL00007	0.014	1.565	9.140	5.833	9.486**	2.454**	<0.001*	<0.001
Bacteria	Bacteroidota	Bacteroidia	Bacteroidales	Dysgonomonadaceae	Dysgonomonas	CL00012	4.462	0.021	0.628	0.672	<0.001**	4.979**	5.029	<0.001**
Bacteria	Pseudomonadota						45.595	69.074	54.611	56.445	0.511*	-0.915**	-1.167**	<0.001
Bacteria	Pseudomonadota	Gammaproteobacteria					45.444	68.838	54.490	56.364	<0.001	-1.189**	-1.65**	<0.001
Bacteria	Pseudomonadota	Gammaproteobacteria	Cardiobacteriales				8.365	0.167	0.212	1.373	-4.035**	<0.001	2.734**	2.724*
Bacteria	Pseudomonadota	Gammaproteobacteria	Cardiobacteriales	Ignatzschineriaceae			8.365	0.167	0.212	1.373	-5.053**	<0.001	3.253**	2.893**
Bacteria	Pseudomonadota	Gammaproteobacteria	Cardiobacteriales	Ignatzschineriaceae	Ignatzschineria		8.365	0.167	0.212	1.373	-4.996**	<0.001	2.99**	3.055**
Bacteria	Pseudomonadota	Gammaproteobacteria	Cardiobacteriales	Ignatzschineriaceae	Ignatzschineria	CL00009	7.073	0.096	0.125	1.052	-5.86**	<0.001	3.433**	3.349**
Bacteria	Pseudomonadota	Gammaproteobacteria	Enterobacterales				36.604	40.157	27.537	29.977	<0.001*	-0.849**	-0.834**	<0.001
Bacteria	Pseudomonadota	Gammaproteobacteria	Enterobacterales	Budviciaceae			0.200	3.587	3.439	3.384	4.204**	<0.001	0.106	<0.001
Bacteria	Pseudomonadota	Gammaproteobacteria	Enterobacterales	Budviciaceae	Pragia		0.053	2.149	2.732	2.648	5.768**	0.295**	<0.001	<0.001
Bacteria	Pseudomonadota	Gammaproteobacteria	Enterobacterales	Budviciaceae	Pragia	CL00013	0.035	1.535	1.847	1.891	5.75**	0.398**	<0.001	<0.001
Bacteria	Pseudomonadota	Gammaproteobacteria	Enterobacterales	Enterobacteriaceae			1.790	3.490	1.406	1.548	<0.001	-0.606*	-0.849*	<0.001
Bacteria	Pseudomonadota	Gammaproteobacteria	Enterobacterales	Enterobacteriaceae	Enterobacter		0.884	1.547	0.644	0.705	<0.001	<0.001*	<0.001*	<0.001
Bacteria	Pseudomonadota	Gammaproteobacteria	Enterobacterales	Enterobacteriaceae	Enterobacter	CL00016	0.884	1.547	0.644	0.705	<0.001	<0.001	-0.972**	<0.001
Bacteria	Pseudomonadota	Gammaproteobacteria	Enterobacterales	Enterobacteriaceae	Rosenbergiella		0.415	1.033	0.179	0.290	<0.001	<0.001**	<0.001*	<0.001
Bacteria	Pseudomonadota	Gammaproteobacteria	Enterobacterales	Erwiniaceae			0.033	2.255	0.271	0.139	3.156**	-2.765**	-3.655**	<0.001
Bacteria	Pseudomonadota	Gammaproteobacteria	Enterobacterales	Erwiniaceae	Pantoea		0.027	1.878	0.218	0.108	3.047**	-2.868**	-4.138**	<0.001
Bacteria	Pseudomonadota	Gammaproteobacteria	Enterobacterales	Erwiniaceae	Pantoea	CL00022	0.023	1.484	0.167	0.083	2.558**	-2.845**	-4.41**	<0.001
Bacteria	Pseudomonadota	Gammaproteobacteria	Enterobacterales	Morganellaceae			34.567	28.199	22.244	24.803	<0.001	<0.001	-0.304	<0.001
Bacteria	Pseudomonadota	Gammaproteobacteria	Enterobacterales	Morganellaceae	Morganella		10.534	19.431	12.867	18.005	<0.001	<0.001	-0.706	<0.001*
Bacteria	Pseudomonadota	Gammaproteobacteria	Enterobacterales	Morganellaceae	Morganella	CL00001	9.331	16.481	9.984	14.619	<0.001	<0.001	-0.659	0.45*
Bacteria	Pseudomonadota	Gammaproteobacteria	Enterobacterales	Morganellaceae	Proteus		5.364	0.678	1.895	1.205	<0.001*	1.329**	<0.001*	<0.001
Bacteria	Pseudomonadota	Gammaproteobacteria	Enterobacterales	Morganellaceae	Proteus	CL00008	5.124	0.611	1.670	1.050	<0.001**	1.473**	<0.001	<0.001
Bacteria	Pseudomonadota	Gammaproteobacteria	Enterobacterales	Morganellaceae	Providencia		17.962	7.733	6.877	5.150	-1.117**	<0.001	-1.095*	<0.001
Bacteria	Pseudomonadota	Gammaproteobacteria	Enterobacterales	Morganellaceae	Providencia	CL00004	5.814	4.606	2.798	2.563	<0.001	-0.707	-1.179**	<0.001
Bacteria	Pseudomonadota	Gammaproteobacteria	Enterobacterales	Morganellaceae	Providencia	CL00006	8.907	0.548	1.813	0.902	<0.001**	1.749**	<0.001	<0.001
Bacteria	Pseudomonadota	Gammaproteobacteria	Enterobacterales	Yersiniaceae			0.010	2.557	0.141	0.073	3.904**	-3.885**	-4.827**	<0.001
Bacteria	Pseudomonadota	Gammaproteobacteria	Enterobacterales	Yersiniaceae	Yersinia		0.007	2.345	0.064	0.026	2.949**	-4.952**	-6.593**	<0.001
Bacteria	Pseudomonadota	Gammaproteobacteria	Enterobacterales	Yersiniaceae	Yersinia	CL00019	0.006	2.174	0.057	0.019	2.693**	<0.001**	<0.001**	<0.001

Bacteria	Pseudomonadota	Gammaproteobacteria	Orbales				0.139	13.967	20.844	21.423	8.322**	<0.001	0.276	<0.001
Bacteria	Pseudomonadota	Gammaproteobacteria	Orbales	Orbaceae			0.139	13.967	20.844	21.423	7.378**	0.76**	0.839**	<0.001
Bacteria	Pseudomonadota	Gammaproteobacteria	Orbales	Orbaceae	Orbus		0.136	13.888	20.656	21.156	7.369**	0.641**	0.389**	<0.001
Bacteria	Pseudomonadota	Gammaproteobacteria	Orbales	Orbaceae	Orbus	CL00002	0.105	11.691	17.584	18.440	7.347**	0.827**	<0.001*	<0.001
Bacteria	Pseudomonadota	Gammaproteobacteria	Pseudomonadales				0.298	14.065	5.765	3.537	5.621**	<0.001**	-2.248**	<0.001
Bacteria	Pseudomonadota	Gammaproteobacteria	Pseudomonadales	Moraxellaceae			0.206	13.954	5.715	3.534	5.045**	-0.985**	-1.642**	<0.001
Bacteria	Pseudomonadota	Gammaproteobacteria	Pseudomonadales	Moraxellaceae	Acinetobacter		0.091	12.900	4.642	2.853	5.67**	-1.25**	-2.212**	<0.001
Bacteria	Pseudomonadota	Gammaproteobacteria	Pseudomonadales	Moraxellaceae	Acinetobacter	CL00005	0.040	9.557	2.102	1.023	5.527**	-1.839**	-3.515**	<0.001

Legend: IL – input larvae

FD – larvae fed by freshly prepared feed

CS-C – larvae fed by feed naturally contaminated with microorganisms and stored in closed containers for 5 days at refrigerated temperature

OS-T – larvae fed by feed naturally contaminated with microorganisms and stored in opened containers for 5 days at room temperature

Table S3: Pairwise comparion of fungal community difference (PERMANOVA)

p values

R2 values		IL	FD	CS-C	OS-T
	IL	NA	0.001	0.001	0.001
	FD	0.2879197	NA	0.833	0.001
	CS-C	0.2989859	0.0271727	NA	0.001
	OS-T	0.2898504	0.4663011	0.4528022	NA

Legend: IL – input larvae

FD – larvae fed by freshly prepared feed

CS-C – larvae fed by feed naturally contaminated with microorganisms and stored in closed containers for 5 days at refrigerated temperature

OS-T – larvae fed by feed naturally contaminated with microorganisms and stored in opened containers for 5 days at room temperature

NA - Not Applicable

Table S4: Differential abundance analysis of Fungi (only members with more than 1% in any variant are listed)

Phylogenetic group						Mean occurence in variant				DeSeq2 Log2fold ratio (*p=<0.05;**p=<0.01)			
phylum	class	order	family	genus	OTU	IL	FD	CS-C	OS-T	IL vs FD	OS-T vs FD	OS-T vsCS-C	FD vsCS-C
Ascomycota						78.43	90.65	85.89	20.58	-0.019**	1.657**	2.759**	0.286
Ascomycota	Dothideomycetes					12.42	4.90	3.45	1.50	-0.756	0.202	-0.095	<0.001
Ascomycota	Dothideomycetes	Cladosporiales				1.92	1.61	0.55	0.70	0.222	-1.205	-2.042**	<0.001
Ascomycota	Dothideomycetes	Cladosporiales	Cladosporiaceae			1.92	1.61	0.55	0.70	<0.001	-1.313	<0.001**	<0.001
Ascomycota	Dothideomycetes	Cladosporiales	Cladosporiaceae	Cladosporium		1.92	1.61	0.55	0.70	<0.001	<0.001**	-4.596**	<0.001
Ascomycota	Dothideomycetes	Cladosporiales	Cladosporiaceae	Cladosporium	CL0013	1.91	1.61	0.47	0.68	<0.001	<0.001**	-4.759**	<0.001
Ascomycota	Dothideomycetes	Pleosporales				9.92	2.82	2.00	0.73	-1.06	-0.603	<0.001	<0.001
Ascomycota	Dothideomycetes	Pleosporales	Didymellaceae			1.22	0.06	0.14	0.01	<0.001	-0.19	<0.001	<0.001
Ascomycota	Dothideomycetes	Pleosporales	Periconiaceae			1.59	0.50	0.40	0.04	<0.001	0.696	<0.001	<0.001
Ascomycota	Dothideomycetes	Pleosporales	Periconiaceae	Periconia		1.59	0.50	0.40	0.04	<0.001	<0.001	<0.001	<0.001
Ascomycota	Dothideomycetes	Pleosporales	Periconiaceae	Periconia	CL0032	1.46	0.36	0.29	0.03	<0.001	<0.001	<0.001	<0.001
Ascomycota	Dothideomycetes	Pleosporales	Phaeosphaeriaceae			1.20	0.34	0.35	0.37	<0.001	-1.841	<0.001	<0.001
Ascomycota	Dothideomycetes	Pleosporales	Phaeosphaeriaceae	Paraphoma		1.14	0.28	0.29	0.36	<0.001	<0.001	<0.001	<0.001
Ascomycota	Dothideomycetes	Pleosporales	Phaeosphaeriaceae	Paraphoma	CL0040	1.13	0.24	0.27	0.36	<0.001	<0.001	<0.001	<0.001
Ascomycota	Dothideomycetes	Pleosporales	Pleosporaceae			2.93	0.34	0.60	0.05	<0.001	0.391	<0.001	<0.001
Ascomycota	Dothideomycetes	Pleosporales	Pleosporaceae	Alternaria		2.68	0.26	0.44	0.04	<0.001	<0.001	<0.001	<0.001
Ascomycota	Dothideomycetes	Pleosporales	Pleosporaceae	Alternaria	CL0036	1.71	0.01	0.03	0.00	<0.001*	<0.001	<0.001	<0.001
Ascomycota	Dothideomycetes	Pleosporales	Pyrenochaetopsidaceae			1.07	0.00	0.02	0.01	<0.001	-0.506	<0.001	<0.001
Ascomycota	Dothideomycetes	Pleosporales	Pyrenochaetopsidaceae	Pyrenochaetopsis		1.07	0.00	0.02	0.01	<0.001	<0.001	<0.001	<0.001
Ascomycota	Eurotiomycetes					17.31	8.72	7.95	0.80	-0.124	2.281**	1.908**	<0.001
Ascomycota	Eurotiomycetes	Eurotiales				15.18	8.24	7.32	0.74	0.163	1.471	<0.001	<0.001
Ascomycota	Eurotiomycetes	Eurotiales	Aspergillaceae			14.09	8.06	7.21	0.44	<0.001	2.327**	<0.001	<0.001
Ascomycota	Eurotiomycetes	Eurotiales	Aspergillaceae	Aspergillus		6.15	3.07	1.65	0.17	<0.001	<0.001	<0.001	<0.001
Ascomycota	Eurotiomycetes	Eurotiales	Aspergillaceae	Aspergillus	CL0020	2.77	0.67	0.75	0.07	<0.001*	<0.001*	<0.001	<0.001
Ascomycota	Eurotiomycetes	Eurotiales	Aspergillaceae	Aspergillus	CL0030	2.01	0.30	0.44	0.05	<0.001**	<0.001**	<0.001	<0.001
Ascomycota	Eurotiomycetes	Eurotiales	Aspergillaceae	Aspergillus	CL0037	0.12	1.89	0.01	0.00	<0.001	<0.001	<0.001	<0.001
Ascomycota	Eurotiomycetes	Eurotiales	Aspergillaceae	Penicillium		7.94	4.99	5.56	0.27	<0.001	<0.001	<0.001	<0.001
Ascomycota	Eurotiomycetes	Eurotiales	Aspergillaceae	Penicillium	CL0009	0.00	3.04	3.75	0.02	<0.001**	<0.001	<0.001	<0.001
Ascomycota	Eurotiomycetes	Eurotiales	Aspergillaceae	Penicillium	CL0021	2.88	0.59	0.63	0.03	-4.963**	<0.001	<0.001	<0.001

Ascomycota	Eurotiomycetes	Eurotiales	Aspergillaceae	Penicillium	CL0034	1.23	0.44	0.20	0.02	<0.001*	<0.001	<0.001	<0.001
Ascomycota	Eurotiomycetes	Eurotiales	Aspergillaceae	Penicillium	CL0044	1.31	0.29	0.22	0.01	<0.001	<0.001	<0.001	<0.001
Ascomycota	Eurotiomycetes	Eurotiales	Trichocomaceae			1.09	0.18	0.10	0.30	<0.001	-0.897	<0.001	<0.001
Ascomycota	Eurotiomycetes	Eurotiales	Trichocomaceae	Talaromyces		1.09	0.18	0.10	0.30	<0.001	<0.001	<0.001	<0.001
Ascomycota	Eurotiomycetes	Chaetothyriales				2.12	0.44	0.52	0.04	-0.815	0.524	<0.001	<0.001
Ascomycota	Eurotiomycetes	Chaetothyriales	Herpotrichiellaceae			1.22	0.36	0.36	0.04	<0.001	0.269	<0.001	<0.001
Ascomycota	Eurotiomycetes	Chaetothyriales	Herpotrichiellaceae	Exophiala		1.22	0.21	0.31	0.03	<0.001	<0.001	<0.001	<0.001
Ascomycota	Leotiomycetes					2.63	1.23	0.68	0.33	-0.193	0.262	-0.358	<0.001
Ascomycota	Leotiomycetes	Helotiales				2.18	0.86	0.60	0.31	-0.261	-0.801	<0.001	<0.001
Ascomycota	Leotiomycetes	Helotiales	Calloriaceae			2.06	0.69	0.36	0.15	<0.001	-0.241	<0.001	<0.001
Ascomycota	Leotiomycetes	Helotiales	Calloriaceae	Populomyces		2.06	0.69	0.36	0.15	<0.001	<0.001	<0.001	<0.001
Ascomycota	Leotiomycetes	Helotiales	Calloriaceae	Populomyces	CL0039	1.25	0.28	0.17	0.01	<0.001*	<0.001	<0.001	<0.001
Ascomycota	Pichiomycetes					17.33	63.31	65.97	5.83	2.059**	2.311**	2.116**	<0.001
Ascomycota	Pichiomycetes	Pichiales				0.58	38.63	38.74	1.05	7.095**	2.668**	3.218**	<0.001
Ascomycota	Pichiomycetes	Pichiales	Pichiaceae			0.58	38.63	38.74	1.05	7.315**	2.259**	2.448**	<0.001
Ascomycota	Pichiomycetes	Pichiales	Pichiaceae	Pichia		0.58	38.63	38.74	1.05	7.265**	1.8**	<0.001	<0.001
Ascomycota	Pichiomycetes	Pichiales	Pichiaceae	Pichia	CL0001	0.23	38.61	38.60	0.99	6.076**	<0.001	1.953**	<0.001
Ascomycota	Pichiomycetes	Serinales				16.75	24.69	27.23	4.78	1.023	0.112	<0.001	<0.001
Ascomycota	Pichiomycetes	Serinales	Debaryomycetaceae			16.75	24.69	27.23	4.78	<0.001	0.142	<0.001	<0.001
Ascomycota	Pichiomycetes	Serinales	Debaryomycetaceae	Diutina		15.12	23.91	26.84	0.29	<0.001	3.353**	1.569**	<0.001
Ascomycota	Pichiomycetes	Serinales	Debaryomycetaceae	Diutina	CL0002	7.82	22.61	26.24	0.23	<0.001	<0.001	<0.001	<0.001
Ascomycota	Pichiomycetes	Serinales	Debaryomycetaceae	Diutina	CL0011	4.63	0.03	0.00	0.01	<0.001**	<0.001	<0.001*	<0.001
Ascomycota	Pichiomycetes	Serinales	Debaryomycetaceae	Diutina	CL0019	2.46	0.88	0.41	0.04	<0.001*	<0.001	<0.001*	<0.001
Ascomycota	Pichiomycetes	Serinales	Debaryomycetaceae	Meyerozyma		0.03	0.01	0.03	1.74	<0.001	<0.001**	<0.001**	<0.001
Ascomycota	Pichiomycetes	Serinales	Debaryomycetaceae	Meyerozyma	CL0025	0.03	0.00	0.01	1.74	<0.001	<0.001**	<0.001**	<0.001
Ascomycota	Pichiomycetes	Serinales	Debaryomycetaceae	Spathaspora		0.17	0.02	0.09	2.73	<0.001	<0.001**	<0.001**	<0.001
Ascomycota	Pichiomycetes	Serinales	Debaryomycetaceae	Spathaspora	CL0014	0.02	0.00	0.04	2.72	<0.001	<0.001**	<0.001**	<0.001
Ascomycota	Saccharomycetes					8.02	3.33	1.09	2.40	-0.573	-1.302	-3.558	<0.001
Ascomycota	Saccharomycetes	Phaffomycetales				0.95	0.29	0.11	2.15	-0.385	-5.376**	<0.001**	<0.001
Ascomycota	Saccharomycetes	Phaffomycetales	Wickerhamomycetaceae			0.95	0.29	0.05	2.14	<0.001	-5.241**	<0.001**	<0.001
Ascomycota	Saccharomycetes	Phaffomycetales	Wickerhamomycetaceae	Wickerhamomyces		0.95	0.29	0.05	2.14	<0.001	<0.001**	<0.001**	<0.001
Ascomycota	Saccharomycetes	Phaffomycetales	Wickerhamomycetaceae	Wickerhamomyces	CL0017	0.95	0.29	0.05	2.14	<0.001	<0.001**	<0.001**	<0.001
Ascomycota	Saccharomycetes	Saccharomycetales				7.06	3.04	0.98	0.25	-0.612	0.712	<0.001	<0.001

Ascomycota	Saccharomycetes	Saccharomycetales	Saccharomycetaceae			7.06	3.04	0.98	0.25	<0.001	0.683	<0.001	<0.001
Ascomycota	Saccharomycetes	Saccharomycetales	Saccharomycetaceae	Saccharomyces		6.33	3.04	0.98	0.25	<0.001	<0.001	<0.001**	<0.001
Ascomycota	Saccharomycetes	Saccharomycetales	Saccharomycetaceae	Saccharomyces	CL0007	6.33	3.04	0.98	0.24	-2.458**	<0.001*	<0.001**	<0.001
Ascomycota	Sordariomycetes					20.40	9.09	6.61	9.15	-0.517	-1.297	-1.465	<0.001
Ascomycota	Sordariomycetes	Glomerellales				1.18	0.51	0.41	0.01	0.251	2.361*	<0.001	<0.001
Ascomycota	Sordariomycetes	Glomerellales	Plectosphaerellaceae			1.18	0.51	0.40	0.01	<0.001	2.32	<0.001	<0.001
Ascomycota	Sordariomycetes	Hypocreales				13.71	5.27	3.73	6.90	-0.487	-2.588	-2.729	<0.001
Ascomycota	Sordariomycetes	Hypocreales	Bionectriaceae			2.29	0.15	0.18	0.04	-3.928*	-0.732	<0.001	<0.001
Ascomycota	Sordariomycetes	Hypocreales	Bionectriaceae	Geosmithia		1.90	0.14	0.01	0.02	<0.001	<0.001*	<0.001**	<0.001
Ascomycota	Sordariomycetes	Hypocreales	Bionectriaceae	Geosmithia	CL0026	1.84	0.00	0.00	0.00	<0.001	NA	NA	NA
Ascomycota	Sordariomycetes	Hypocreales	Cordycipitaceae			1.62	0.11	0.23	2.56	<0.001	-5.7	<0.001	<0.001
Ascomycota	Sordariomycetes	Hypocreales	Cordycipitaceae	Beauveria		1.62	0.11	0.23	2.56	<0.001	<0.001	<0.001	<0.001
Ascomycota	Sordariomycetes	Hypocreales	Cordycipitaceae	Beauveria	CL0015	1.41	0.07	0.21	2.55	<0.001	<0.001*	<0.001	<0.001
Ascomycota	Sordariomycetes	Hypocreales	Hypocreaceae			1.18	0.03	0.04	0.02	<0.001	-0.3	<0.001	<0.001
Ascomycota	Sordariomycetes	Hypocreales	Hypocreaceae	Trichoderma		1.18	0.03	0.04	0.02	<0.001	<0.001	<0.001	<0.001
Ascomycota	Sordariomycetes	Hypocreales	Nectriaceae			7.93	4.42	2.91	4.25	<0.001	-2.34	-3.089*	<0.001
Ascomycota	Sordariomycetes	Hypocreales	Nectriaceae	Dactylonectria		0.03	0.00	0.00	3.25	<0.001	<0.001**	<0.001**	<0.001
Ascomycota	Sordariomycetes	Hypocreales	Nectriaceae	Dactylonectria	CL0018	0.01	0.00	0.00	3.24	<0.001	<0.001*	<0.001*	NA
Ascomycota	Sordariomycetes	Hypocreales	Nectriaceae	Fusarium		7.32	4.40	2.85	0.67	<0.001	<0.001	<0.001**	<0.001
Ascomycota	Sordariomycetes	Hypocreales	Nectriaceae	Fusarium	CL0016	0.01	1.00	1.86	0.02	<0.001	<0.001	<0.001	<0.001
Ascomycota	Sordariomycetes	Hypocreales	Nectriaceae	Fusarium	CL0023	2.27	0.73	0.39	0.08	<0.001*	<0.001	<0.001	<0.001
Ascomycota	Sordariomycetes	Hypocreales	Nectriaceae	Fusarium	CL0027	2.53	0.00	0.00	0.00	<0.001	NA	NA	NA
Ascomycota	Sordariomycetes	Hypocreales	Nectriaceae	Fusarium	CL0048	0.00	1.21	0.00	0.00	<0.001	<0.001	NA	<0.001
Ascomycota	Sordariomycetes	Ophiostomatales				1.38	0.50	0.44	1.50	-0.206	-3.21**	-4.089**	<0.001
Ascomycota	Sordariomycetes	Ophiostomatales	Ophiostomataceae			1.38	0.50	0.44	1.50	<0.001	-2.934**	<0.001**	<0.001
Ascomycota	Sordariomycetes	Ophiostomatales	Ophiostomataceae	Ophiostoma		1.00	0.34	0.35	1.50	<0.001	-4.186**	<0.001**	<0.001
Ascomycota	Sordariomycetes	Ophiostomatales	Ophiostomataceae	Ophiostoma	CL0024	0.50	0.28	0.04	1.39	<0.001	<0.001**	<0.001**	<0.001
Ascomycota	Sordariomycetes	Sordariales				2.78	1.75	1.31	0.58	0.112	-0.814	<0.001*	<0.001
Basidiomycota						12.93	5.26	3.99	4.67	-0.61*	0.156	0.122	-0.038
Basidiomycota	Agaricomycetes					2.12	1.34	1.09	1.83	0.602	-1.076	-2.013**	<0.001
Basidiomycota	Agaricomycetes	Polyporales				0.60	0.16	0.14	1.69	0.081	-5.001**	-5.813**	<0.001
Basidiomycota	Agaricomycetes	Polyporales	Irpicaceae			0.01	0.04	0.01	1.54	<0.001	-7.473**	-9.212**	<0.001
Basidiomycota	Agaricomycetes	Polyporales	Irpicaceae	Meruliopsis		0.01	0.04	0.01	1.54	<0.001	-8.33**	-11.385**	<0.001

Basidiomycota	Agaricomycetes	Polyporales	Irpicaceae	Meruliopsis	CL0029	0.01	0.04	0.01	1.54	<0.001	-10.679**	<0.001**	<0.001
Basidiomycota	Exobasidiomycetes					1.52	0.55	0.70	0.12	-0.185	0.681	1.119	<0.001
Basidiomycota	Exobasidiomycetes	Microstromatales				1.52	0.55	0.70	0.12	-0.453	0.002	<0.001	<0.001
Basidiomycota	Exobasidiomycetes	Microstromatales	Microstromataceae			1.52	0.55	0.70	0.12	<0.001	-0.045	<0.001	<0.001
Basidiomycota	Exobasidiomycetes	Microstromatales	Microstromataceae	Microstroma		1.52	0.55	0.70	0.12	<0.001	<0.001	<0.001*	<0.001
Basidiomycota	Exobasidiomycetes	Microstromatales	Microstromataceae	Microstroma	CL0031	1.52	0.55	0.70	0.12	<0.001	<0.001*	<0.001	<0.001
Basidiomycota	Malasseziomycetes					6.42	2.34	1.34	2.52	-0.571	-1.565*	-3.276*	<0.001
Basidiomycota	Malasseziomycetes	Malasseziales				6.42	2.34	1.34	2.52	-0.52	-3.379*	-3.632*	<0.001
Basidiomycota	Malasseziomycetes	Malasseziales	Malasseziaceae			6.42	2.34	1.34	2.52	<0.001	-2.68**	-3.385**	<0.001
Basidiomycota	Malasseziomycetes	Malasseziales	Malasseziaceae	Malassezia		6.42	2.34	1.34	2.52	<0.001	-4.562**	-6.264**	<0.001
Basidiomycota	Malasseziomycetes	Malasseziales	Malasseziaceae	Malassezia	CL0010	4.63	0.99	0.48	0.05	-4.055**	<0.001	<0.001	<0.001
Basidiomycota	Malasseziomycetes	Malasseziales	Malasseziaceae	Malassezia	CL0022	0.03	0.00	0.01	1.93	<0.001	<0.001**	<0.001**	<0.001
Basidiomycota	Tremellomycetes					2.42	0.71	0.75	0.15	-0.765	0.77	0.773	<0.001
Basidiomycota	Tremellomycetes	Filobasidiales				1.76	0.29	0.48	0.14	-1.35	-0.954	<0.001	<0.001
Basidiomycota	Tremellomycetes	Filobasidiales	Filobasidiaceae			1.29	0.27	0.47	0.14	<0.001	-1.079	<0.001	<0.001
Basidiomycota	Tremellomycetes	Filobasidiales	Filobasidiaceae	Filobasidium		1.27	0.16	0.47	0.09	<0.001	<0.001	<0.001	<0.001
Basidiomycota	Tremellomycetes	Filobasidiales	Filobasidiaceae	Filobasidium	CL0042	1.27	0.16	0.42	0.09	<0.001**	<0.001*	<0.001	<0.001
Mucoromycota						8.64	4.09	10.12	74.74	-0.37	-4.561**	-2.8**	1.166
Mucoromycota	Mucoromycetes					7.84	4.01	9.99	74.71	0.076	-5.143**	-4.028**	<0.001
Mucoromycota	Mucoromycetes	Mucorales				7.84	4.01	9.99	74.71	0.544	-6.347**	-3.84**	2.299
Mucoromycota	Mucoromycetes	Mucorales	Mucoraceae			7.34	3.75	4.48	61.66	<0.001	-6.356**	-6.147**	<0.001
Mucoromycota	Mucoromycetes	Mucorales	Mucoraceae	Actinomucor		3.26	0.02	0.04	0.02	<0.001	<0.001	<0.001	<0.001
Mucoromycota	Mucoromycetes	Mucorales	Mucoraceae	Actinomucor	CL0028	2.91	0.00	0.00	0.00	<0.001	<0.001	NA	<0.001
Mucoromycota	Mucoromycetes	Mucorales	Mucoraceae	Mucor		4.08	3.73	4.44	61.65	<0.001	-7.312**	-8.466**	<0.001
Mucoromycota	Mucoromycetes	Mucorales	Mucoraceae	Mucor	CL0003	0.63	1.84	0.76	42.46	<0.001	<0.001**	<0.001**	<0.001
Mucoromycota	Mucoromycetes	Mucorales	Mucoraceae	Mucor	CL0004	0.15	0.58	0.36	18.82	<0.001	<0.001**	<0.001**	<0.001
Mucoromycota	Mucoromycetes	Mucorales	Mucoraceae	Mucor	CL0033	0.00	0.08	2.97	0.00	<0.001	<0.001	<0.001	<0.001
Mucoromycota	Mucoromycetes	Mucorales	Mucoraceae	Mucor	CL0043	1.42	0.79	0.11	0.00	<0.001	<0.001	<0.001	<0.001
Mucoromycota	Mucoromycetes	Mucorales	Rhizopodaceae			0.50	0.26	5.51	13.05	<0.001	-7.093**	<0.001**	6.042
Mucoromycota	Mucoromycetes	Mucorales	Rhizopodaceae	Rhizopus		0.50	0.26	5.51	13.05	<0.001	-7.997**	<0.001**	5.35
Mucoromycota	Mucoromycetes	Mucorales	Rhizopodaceae	Rhizopus	CL0005	0.50	0.26	5.38	13.05	<0.001	<0.001**	<0.001**	<0.001

Legend: IL – input larvae

FD – larvae fed by freshly prepared feed

CS-C – larvae fed by feed naturally contaminated with microorganisms and stored in closed containers for 5 days at refrigerated temperature

OS-T – larvae fed by feed naturally contaminated with microorganisms and stored in opened containers for 5 days at room temperature

NA - Not Applicable
